# Innovative design of 3D-printed nasopharyngeal pediatric swab for COVID-19 detection

**DOI:** 10.1186/s41205-021-00113-9

**Published:** 2021-08-20

**Authors:** Ameerah Alazemi, Ghadeer AbdulHussain, Abdullah Alawwam, Ali Al-Shatti, Mohammad Alghounaim, Sulaiman Almazeedi, Sarah Al Youha, Salman Al-Sabah

**Affiliations:** 1 Microbiology Department, Faculty of Medicine, Kuwait University, Kuwait City, Kuwait; 2 Microbiology Department, Faculty of Science, Kuwait University, Kuwait City, Kuwait; 3 Biological Sciences Department, Faculty of Science, Kuwait University, Kuwait City, Kuwait; 4 Kuwait Integrated Petroleum Industries Company (KIPIC), Salmiya, Kuwait; 5 Department of Pediatrics, Amiri Hospital, Ministry of Health, Kuwait City, Kuwait; 6 Department of Surgery, Jaber Al-Ahmad Hospital, Ministry of Health, Kuwait City, Kuwait

**Keywords:** COVID19, Three-dimensional printing, Nasal swabs, Pediatric, Coronavirus

## Abstract

3-dimensional (3D) printing technology provides a solution to meet the high demand for producing adult nasal swabs. A smaller, more flexible nasopharyngeal swab needs to be developed for children and infants suspected of having coronavirus. The information shared here presents a novel 3D-printed pediatric swab for the purpose of collecting upper respiratory clinical specimens.

## Introduction

On January 30th 2020, the World Health Organization declared severe acute respiratory syndrome-related coronavirus 2 (SARS-CoV-2), a novel Betacornovrius responsible for causing coronavirus disease 2019 (COVID-19) to be a global pandemic [1]. The rapid escalation and spread of the virus with more than 100 million confirmed cases and more than two million confirmed deaths worldwide, emphasized the importance of employing accurate diagnostic tests and results to hinder the viral transmission [2]. Consequently, shortages in crucial medical supplies such as ventilators, masks, face shields, nasopharyngeal (NP) swabs, and others have impacted all countries [3, 4].

Epidemiologic studies have demonstrated that the elderly population is more prone to infection by coronavirus [5]. The prevalence of COVID-19 in the pediatric population is low [6]; infants [7] and children affected with SARS-CoV-2 exhibit milder symptoms, lower hospitalization, and mortality rates [8, 9]. However, patients with comorbidities are inclined to develop severe acute disease [10–12].

Detection of SARS-CoV-2 on respiratory specimens collected by nasopharyngeal (NP) swabs is the common modality for sample collection and subsequent SARS-CoV-2 detection by reverse transcription polymerase chain reaction (RT-PCR) [13]. To combat the global deficit of commercial (NP) swabs, 3-dimentionally (3D)-printed NP swabs have been created and clinically validated as an alternative to the standard NP swab [14–16]. However, the majority of 3D-printed NP swabs are designed for adults, which can be too rigid and large for safe pediatric use [17]. The main basis for the diagnosis of COVID-19 in children is the RT-PCR detection of SARS-CoV-2 in nasopharyngeal samples [13]. This issue accentuated the need to design a pediatric NP swab with qualities such as efficient collection of viral particles from the posterior nasopharynx and flexibility to maneuver inside the nasopharynx.

The nasopharyngeal area has a high expression of angiotensin-converting enzyme 2 (ACE 2) receptors making it the ideal swabbing site. For adults, 8–10 cm is the typical distance from the nostril to the nasopharynx posterior wall. On the other hand, infants and children have a shorter nasal cavity with 6–7 cm being the typical distance from the nostril cavity to the nasopharynx posterior wall [18]. That’s why we were meticulous in choosing clinically acceptable lengths for children aged 1–3 years. Various databases such as: Access Pediatrics, Scopus, PubMed, EMBASE, Web of Science, and The Cochrane Library were used to conduct extensive review of literature to comprehend the optimal characteristics of a pediatric NP swab. Additionally, a panel of experts from science and medical fields including medical virology, pediatric otolaryngology, registered and trained nurses, medical laboratory clinicians, and engineers with in-depth knowledge in 3D printing technologies were consulted to provide their opinion regarding the attributes of the NP swab. Based on this, a list of traits was consented upon for designing the prototype for the NP swab (Table 1).

**Table 1:** Ideal characteristics of pediatric NP swab prototype based on literature review and expert panel consensus

**Medical properties**
◾ Rigid, non-brittle to accommodate the pressure and sufficiently collect an upper respiratory sample.
◾ Flexible handle to maneuver inside the nasopharynx while maintaining tensile strength.
◾ Highly absorbent tip composed of polyester to collect and retain fluid.
◾ Adequate sterilization ought to not adversely impact the swabs’ integrity.
**Dimensions**
◾ Ideal length 10-12 centimeters to reach the posterior nasopharynx.
◾ A rounded smooth tip to prevent trauma to the surrounding tissue.
**Biocompatibility and biosafety**
◾ Non-toxic material.
◾ Does not interfere with PCR reagents.

## Three-dimensional printer description and nasopharyngeal swab procedure

Fused deposition modeling (FDM) 3D printer (Prusa™ I3 MK2S) was used in the production of the pediatric nasopharyngeal swabs. The printer was available along with polylactic acid filament purchased from two different brands Hatchbox and Esun. Prototypes were done using Simplify3d and Fusion360 as the chosen slicing and computer-aided design (CAD) software, respectively. For a standard 0.4 mm nozzle diameter, the layer height was set for 0.2 mm, extrusion width 0.4 mm, with 100 % infill and no support. With the cooling fan switched ON, the bed and extrusion temperature were set for 60 and 200 °C, respectively, at speed 3200–3600 mm/min.

**Fig. 1:**
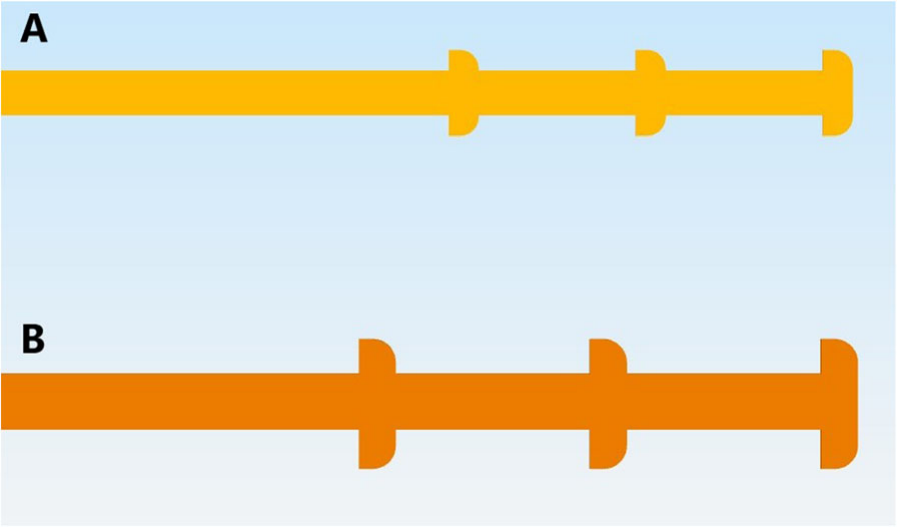
Three-dimensionally designed nasopharyngeal swab for pediatrics (**A**) and adults (B) in which the tip of the swab has three pins/nubs

**Fig. 2:**
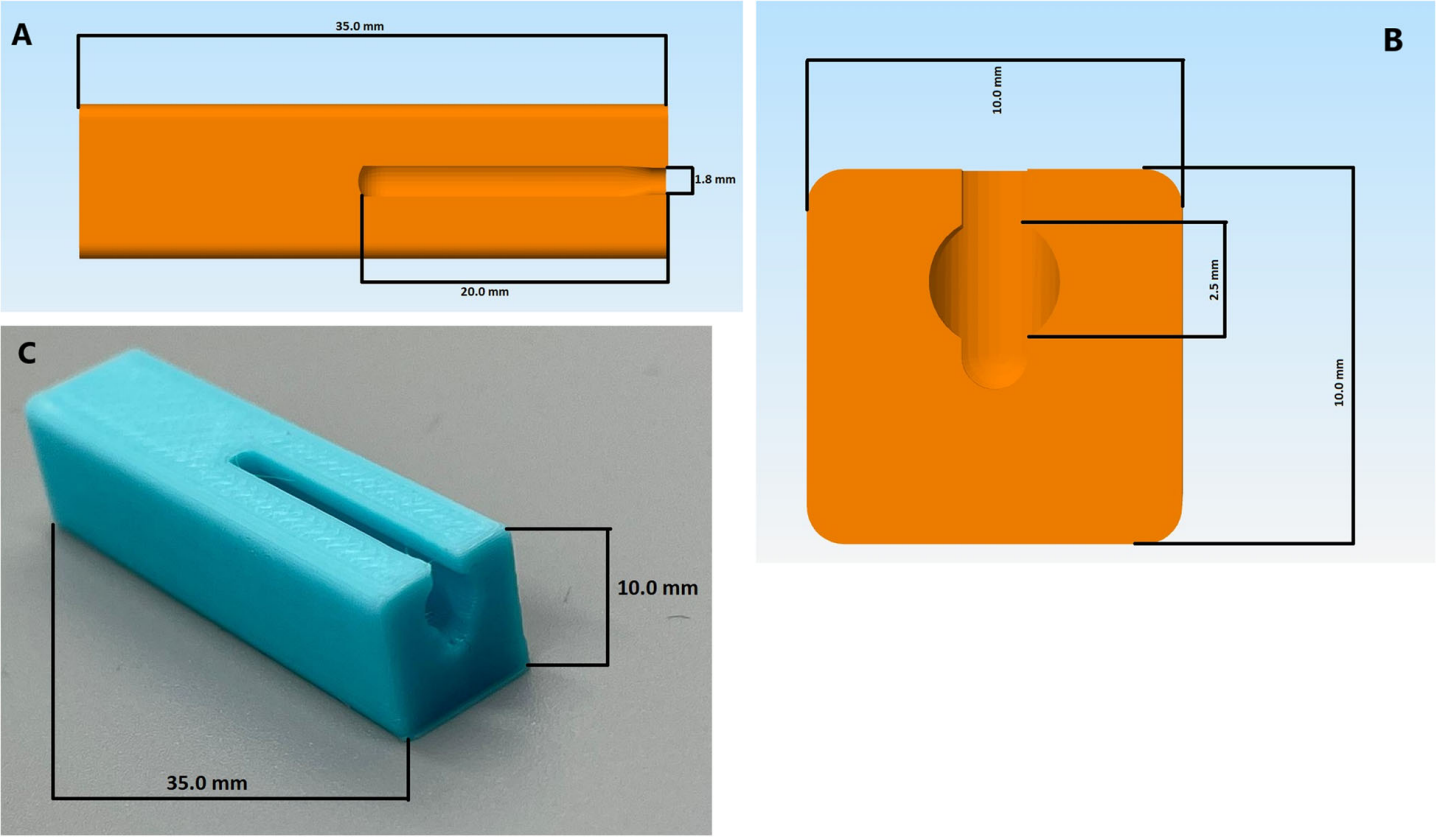
Blueprint model of polyester tip applicator complete (**A**) and top (**B**) view. (**C**) Three-dimensionally printed polyester tip applicator used for assembly

An FDM 3D printer was used because it is a relatively inexpensive equipment (approximately US$ 500) that can be readily purchased [19]. Polylactic acid (PLA) was the chosen material to create swabs because of its safety, biocompatibility, low cost, and resistance to breakage [20–22]. To facilitate production without using adhesive material, the tip of the PLA swab featured small nubs (Fig. 1) to tightly intertwine the polyester threads and decrease the possibility of it becoming unwound. A swab applicator (Fig. 2) was used (https://www.thingiverse.com/thing:4373981) to intertwine the polyester fibers on the tip of the swab [23]. By inserting the PLA swab and some polyester in the applicator, the swab was rotated like a pencil sharpener, ensuring that the polyester was securely wrapped on the tip without the concern of it becoming dislodged, enforced uniformity in the production, and created a brush-like texture. Polyester was chosen because it is chemically safe, does not interfere with PCR reagents, has a high absorption capacity, and elutes into the viral transport media (VTM) [24, 25].

**Fig. 3:**
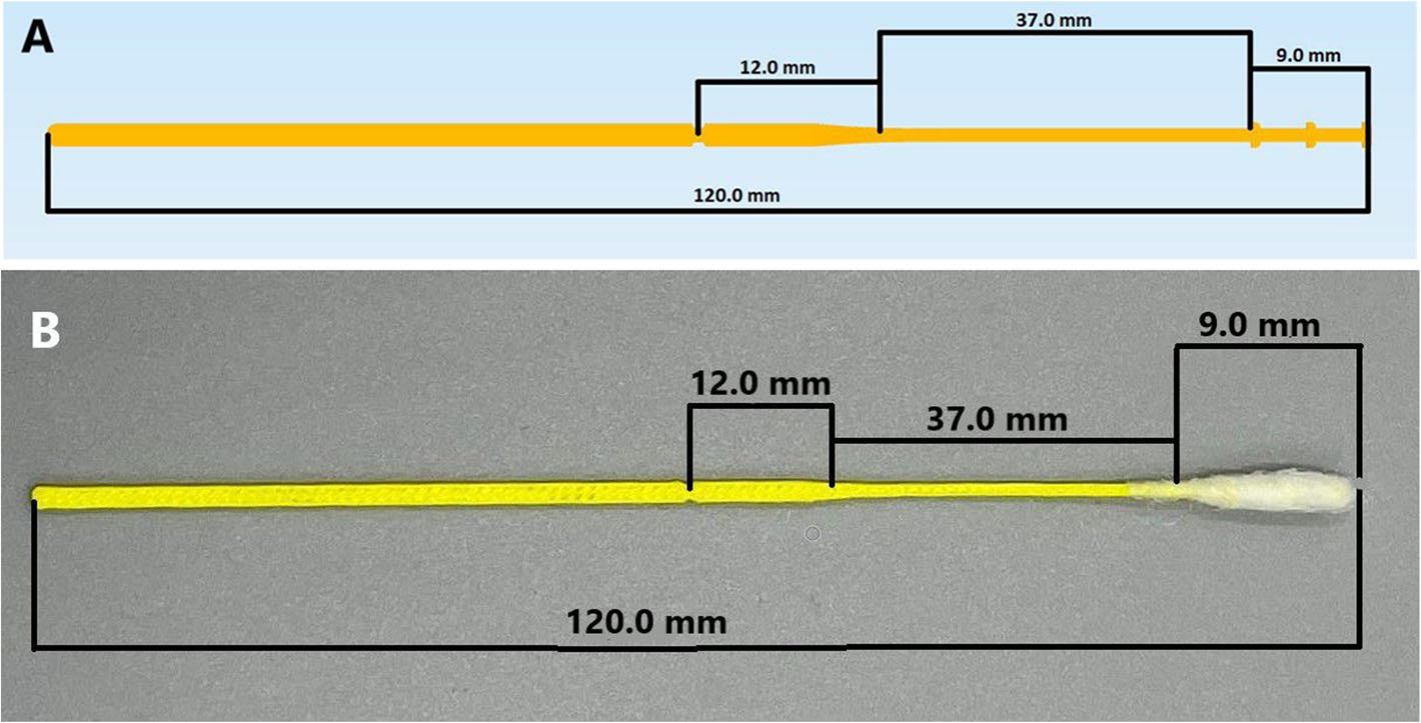
**A** Design of the three- dimensionally paediatric swab. **B** Assembled three- dimensionally printed paediatric swab

**Fig. 4:**
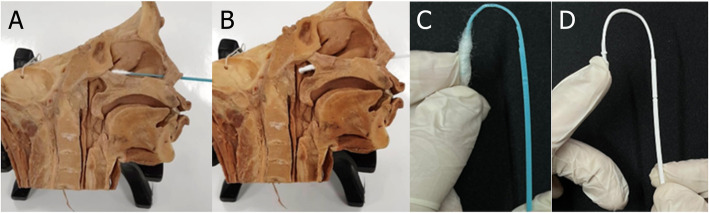
Plastinated nasopharyngeal model with the pediatric swab (**A**) and adult swab (**B**) inside the nasopharynx. Pediatric (**C**) and adult swab (**D**) 180° bending test

**Fig. 5:**
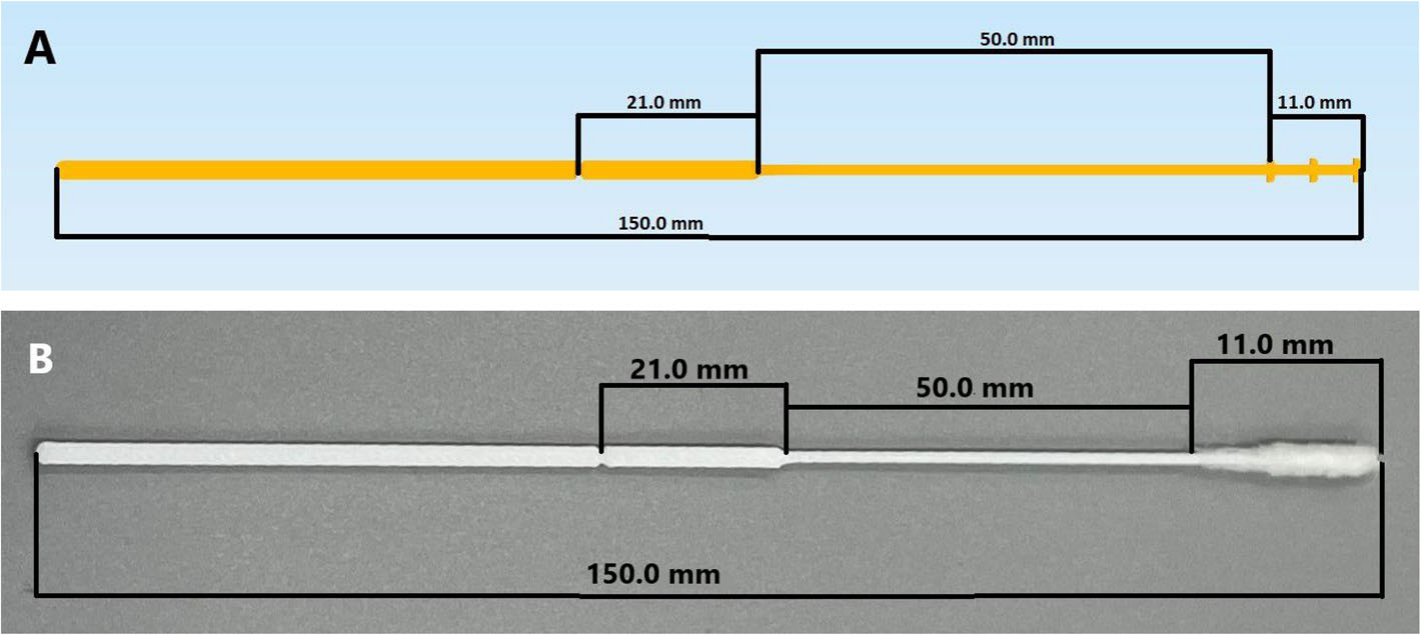
**A** Design of the three-dimensionally adult swab. **B** Assembled three- dimensionally printed adult swab

The final 3D printed pediatric swab (Fig. 3) is 120.0 mm in length with a 94 mm break point (Fig. 3) from the tip. The tip of the swab is 9.0 mm long with three pins per side to adhere the polyester fibers (less than 0.01 g) to the swab, eliminating the need for adhesive substrates. The shaft has 1 mm diameter and is 37.0 mm long. Plastinated nasopharyngeal models from the dissection room in the Anatomy Department, Faculty of Medicine, Kuwait University, were used as a simulation to demonstrate the flexibility of both pediatric and adult swabs in reaching the posterior nasopharynx (Fig. 4). The same swab design with variant measurements was validated for the adults (Fig. 5) [26].

**Fig. 6:**
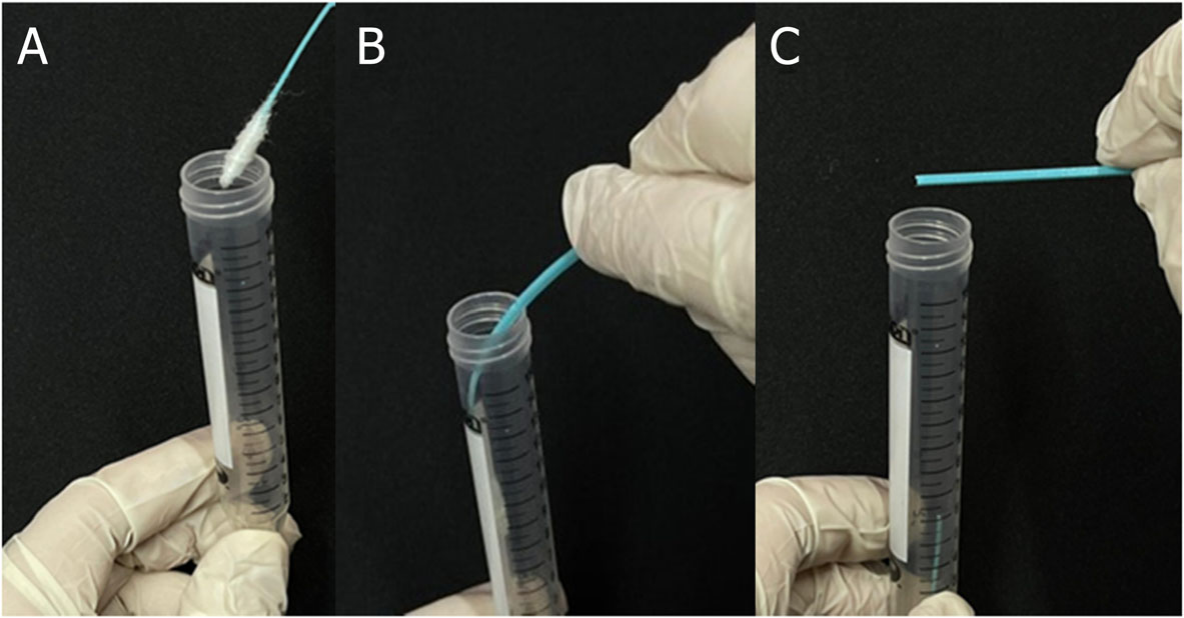
**A** Pediatric swab inserted in VTM tube. **B** The swab inserted in the tube while keeping the breakpoint near the rim of the tube, applicator is broken from the break point indicator line. **C** The swab tip submerged in the VTM tube

In terms of comparisons, the shape and type of material used on the swab tip represent the disparate differences between our 3D printed swab and the existing commercial swab. Our 3D swabs have a solid tip featuring pins that create grooves enabling the polyester to adhere to the tip without using adhesives. Commercial swabs typically employ nylon on the tip using the flocking method, in which the nylon is deposited onto an adhesive coated surface. On the contrary, our 3D swabs can dispense with adhesives and the polyester is fixed on the tip using our 3D printed polyester tip applicator (Fig. 2). It functions as an assembly tool that intertwines the polyester filaments between the pins on the tip. Additionally, after sampling is completed and the swab is inserted in the VTM tube (Fig. 6), the ridges between the pins ensure that the manually wrapped polyester threads will not dislodge. It is possible that the presence of loose synthetic fibers in the viral transport media (VTM) tube may adversely affect downstream viral testing. Thereby, with the use of our 3D printed polyester applicator, the fibers become tightly intertwined between the ridges decreasing the chance of becoming unwound in the VTM tube and of course the nasopharynx. In comparison to the adult version (Fig. 5), the 3D printed pediatric swabs are shorter in length and more flexible, ultimately providing comfort to the patients. The altered dimensions of the pediatric swab and its flexibility will reduce the number of attempts required to reach the posterior nasopharynx and decrease the duration needed to obtain a sample from pediatric patients.

The swabs were sterilized at the Central Sterilization Service Department (CSSD) in Kuwait’s Ministry of Health via low-temperature (45 °C) hydrogen peroxide plasma. Different sterilization techniques have been reported to induce micro and macrostructural changes to the polyester [27, 28]. However, low temperature hydrogen peroxide sterilization uses evaporated hydrogen peroxide at 45 °C, deeming it suitable for heat- labile material. By using hydrogen peroxide vapor at 45 °C, we can ensure the integrity of the polyester will not be compromised during the sterilization process. To confirm adequate sterilization, ten swabs were randomly selected and cultured on blood agar (BAP) plus chocolate agar and incubated at 35 °C ± 2 °C for 48 h. The sterility of the prepared VTM was validated by randomly selecting three tubes from the beginning, middle, and end of each batch, and 100 µL was inoculated on BAP and incubated at 35 ± 2 °C for 48 h. The VTM was concocted at Jaber Al Ahmad Al Sabah Hospital laboratory, Kuwait, according to the protocol published by the Centers for Diseases Control and Prevention (CDC) [29]. Along with the prepared VTM, the 3D printed swab was packed and labeled as a pediatric COVID-19 testing kit.

## Benefits and limitations

The production of pediatric prototype NP swabs using 3D-FDM printers has several advantages. Inexpensive readily available materials were used to create pediatric NP swabs (costs less than US$0.05), making it a reasonable option for countries suffering from a shortage of commercial pediatric swabs. The universal availability of 3D printers and the simplicity of iterating and testing various designs of swabs are attractive factors for producing novel 3D-printed swabs. Nonetheless, for large-scale production, 3D printers are quite slow, approximately 3 h and 15 min to manufacture 60 NP swabs from one printer. Roughly one hour to produce 20 NP swabs, i.e., 3 min/swab using Prusa i3 MK2S.

This brief report has several limitations. The NP swabs were not tested on pediatric subjects, they ought to be compared to commercially manufactured swabs for effective nasopharyngeal sample collection. The swab is designated for a limited age range 1–3 years. Broader age ranges concurrent with iterating different prototypes would make this study more inclusive and rigorous. Another possible limitation would be the type of material used on the swab head. The CDC endorsed the use of nylon or rayon [30], whereas WHO recommended the use of cotton [31]. All recommended options are valid; however, polyester is used in other virology specimen collection systems [25]. Polyester also has high specimen collection and retention in 3D printed adult nasal swabs employed to detect COVID-19 infection [26]. The same principle was applied when opting for polyester on the pediatrics swabs.

A superior alternative to using filament (FDM) printers and polylactic acid filament (PLA) would be using stereolithography (SLA) printers and a medical-grade biocompatible resin specifically formulated for human skin and mucosal membrane. Additionally, produced end-products have a cleaner finish compared to the rough finish observed on products printed by FDM. The downside is SLA printers and the required materials are costly and slower in terms of production rate [19]. Although the value of the printer and material may not be neceassirly major issues, in a pandemic, expenses become amplified because the attention becomes fixated on a few items. In COVID-19, swabs became significantly meagre and the need to purchase them hastily imposed a critical problem. Even though PLA swabs are not composed of medical-grade material, they can do the task efficiently, rapidly, and most importantly safely. Thus, overcoming the issue of expense by using affordable readily-available printers and materials.

## Data Availability

A swab applicator 3D design is available on (https://www.thingiverse.com/thing:4373981). The swab design blueprint and production standard operating procedures (SOPs) are available online (https://projectjaber.com/).

## Abbreviations

3D: 3-dimension
ACE 2: Angiotensin-converting enzyme 2
SARS-CoV-2: Severe acute respiratory syndrome-related coronavirus 2
NP: Nasopharyngeal
COVID-19: Coronavirus disease 2019
RT-PCR: Reverse transcription polymerase chain reaction
FDM: Fused deposition modeling 3D printer
US$: United States dollar (currency)
PLA: Polylactic acid
VTM: Viral transport media
mm: Millimeter (unit of length in the metric system)
°C: Temperatures in Celsius degree
CSSD: Central Sterilization Service Department
BAP: Blood agar
CDC: Centers for Diseases Control and Preventio
